# Transmissibility of the Influenza Virus during Influenza Outbreaks and Related Asymptomatic Infection in Mainland China, 2005-2013

**DOI:** 10.1371/journal.pone.0166180

**Published:** 2016-11-23

**Authors:** Tao Chen, Tianmu Chen, Ruchun Liu, Cuiling Xu, Dayan Wang, Faming Chen, Wenfei Zhu, Xixing Zhang, Jing Yang, Lijie Wang, Zhi Xie, Yongkun Chen, Tian Bai, Yelan Li, Zhiyu Wang, Min Zhang, Shuilian Chen, Yuelong Shu

**Affiliations:** 1 National Institute for Viral Disease Control and Prevention, Collaboration Innovation Center for Diagnosis and Treatment of Infectious Diseases, Chinese Center for Disease Control and Prevention; Key Laboratory for Medical Virology, National Health and Family Planning Commission, Beijing 102206, P.R. China; 2 Department of Malaria, National Institute of Parasitic Diseases, Chinese Center for Disease Control and Prevention, Shanghai, P.R. China; 3 Changsha Center for Disease Control and Prevention, Changsha, P.R. China; University of Hong Kong, HONG KONG

## Abstract

We collected 2768 Influenza-like illness emergency public health incidents from April 1, 2005 to November 30, 2013reported in the Emergency Public Reporting System. After screening by strict inclusion and exclusion criteria, there were 613 outbreaks analyzed with susceptible–exposed–infectious/asymptomatic–removed model in order to estimate the proportion of asymptomatic individuals (*p*) and the effective reproduction number (*R*_*t*_). The relation between *R*_*t*_ and viral subtypes, regions, outbreak sites, populations, and seasons were analyzed. The mean values of *p* of different subtypes ranged from 0.09 to 0.15, but could be as high as up to 0.94. Different subtypes, provinces, regions, and sites of outbreak had statistically significantly different *R*_*t*_. In particular, the southern region also manifested different *R*_*t*_ by affected population size and seasonality. Our results provide China and also the rest of the world a reference to understand characteristics of transmission and develop prevention and control strategies.

## Introduction

Influenza is a respiratory infectious disease which can result in annual infection of 5–15% of the population, leading to 250,000 and 500,000 deaths[1]. Surveillance of the disease agents at the human and animal interface facilitates early detection of influenza transmission. Numerous outbreaks of influenza/ILI are reported every year throughout China. From April 1, 2005 to November 30, 2013, there were 2,768 influenza/Influenza-like illness (ILI) outbreaks recorded in the Emergency Public Reporting System (EPRS).There are two systems in China for influenza surveillance: one is National Public Health Information System and the other is the National Sentinel Surveillance System for ILI. The two systems are linked, such that the influenza outbreak report in the first system is directly synchronized to the second system. Before November 2012 it is mandatory to report any incident with more than 30 cases of ILI weekly in the same unit (equivalent to class IV public health emergency). Since November 2012, the number has been changed from 30 to 10. This study adopted the definition of 30 cases for analysis.

To control outbreaks in schools or work place, the transmissibility of influenza and the proportion of asymptomatic infections are the key factors that should be taken into consideration of control strategies. There are two main categories responding to large-scale contagious outbreaks at city-, province- or region-scales: drug and non-drug intervention, for examples, antiviral treatment, prevention medication, vaccination; and advocate of social contact avoidance, school closure, and travel restrictions respectively. According to "National Health and Family Planning Commission of the People's Republic of China. Guidelines for Dispose of Influenza-like Illness Outbreak (2012th edition), 2012. (in Chinese)", the principal measure to contain school outbreaks is isolation. However, in the presence of asymptomatic infections, isolation can be inefficient because cases are not easy to be identified. Isolation can also become difficult when transmission is intense.

An England cohort study showed that the proportion of influenza asymptomatic infection can be as high as 77 percent [2], while Yang [3] and Longini [4, 5] obtained the estimate of only 33% in North American or Thai population. Therefore, characterization of the transmissibility and the proportion of asymptomatic infection of influenza virus shall provide a reference for policy making and evaluation of public health measures.

Ordinary differential equation (ODE) modeling was first applied to infectious disease modeling in 1927 by Kermack and McKendrick, who established the susceptible–infectious–removed (SIR) model [6]. ODE models have undergone continuous improvement and have been frequently used in mathematical modeling in influenza studies. For example, Stone et al. [7] and Dushoff et al. [8] used SIR and susceptible–infectious–removed–susceptible (SIRS) models, respectively, to simulate the seasonality and periodicity of influenza epidemics. Arino et al. [9] established the SEIAR model of influenza pandemics and simulated the effects of targeted prevention and control measures.

## Materials and Methods

### Data collection criteria

**Fig 1:**
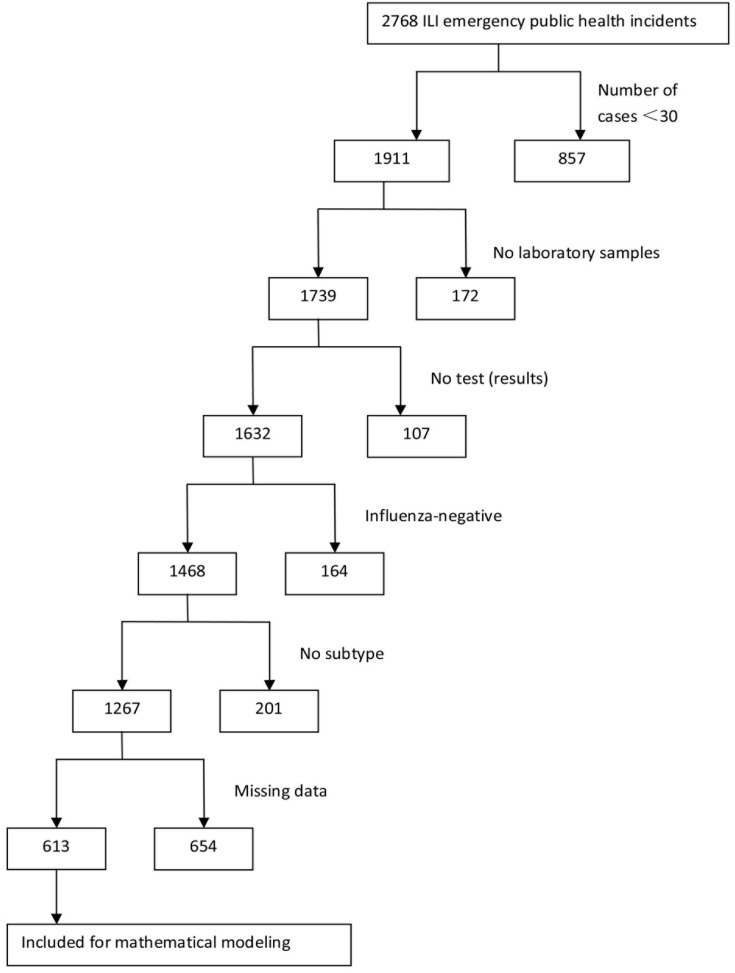
Flowchart of influenza outbreak data inclusion criteria in this study.

Information related to influenza outbreaks reported in China through the EPRS from April 1, 2005 to November 30, 2013 was collected. Influenza-like illness (ILI) refers to a fever (axillary temperature ≥38°C) accompanied by coughing or sore throat and a lack of a laboratory-confirmed diagnosis of the specific pathogen [10, 11]. The criteria for an influenza outbreak was defined as ≥30 ILIs occurring in the same school, preschool, or other collective organization within 1 week [10], with laboratory-confirmed influenza viruses through virus isolation or real-time reverse transcriptase polymerase chain reaction (RT-PCR) analysis. Subjects were included when the following inclusion criteria were met: 1) emergency public health incidents with ≥30 patients; 2) incidents with specific influenza subtypes verified by pathogen examinations including virus isolation and/or nucleic acid detection; and 3) a time interval between the first case and healthcare authority intervention of ≥5 days. The inclusion criteria are summarized as a flowchart in Fig 1.

### Structure of the data and target data for modeling

We used each included outbreak as a unit and collected the names, reported areas, provinces, regions (southern or northern), time of occurrence, accumulated cases, location types of the incidents, number of people affected by a single incident, and laboratory test results of the incidents. We sorted the time distribution of the disease cases of each outbreak within the time interval between the date of onset in the first case and the date of disease control authority intervention as the epidemic outbreak data during a period of no intervention. The method of data capture in the no-intervention period in each outbreak is shown in Figure A in S2 File in the target data section.

### Subtype of influenza

Based on the characteristics of the influenza viruses, we classified the influenza viruses as A (H3N2), A (H1N1) pdm, A (H1N1), B and mixed types. The mixed type refers to a mixture of ≥2 virus types in one case sample detected in a single outbreak; it can include ≥2 subtypes of type A or ≥1 subtype of type A in addition to type B influenza viruses.

### The period between disease onset and recovery

We collected the disease course data from 8 outbreaks between January 1, 2011 and November 30, 2013, totaling 283 cases. The dataset contained the information of location, results of PCR test for influenza virus, the number of cases and the dates of disease onset and recovery. The infectious individual removal coefficient *γ* could then be obtained by taking the inverse of the period between disease onset and recovery. (S1 File).

### Model establishment

We used an ODE model to estimate the effective reproduction number (*R*_*t*_) of outbreak data recorded in EPRS and analyzed the differences in *R*_*t*_ by influenza viruses of various subtypes, areas, provinces, outbreak location types, initial susceptible population sizes, years, and seasons. Previous applications of ODE model to estimate *R*_*t*_ can be found in the work of Sertsou et al [12] and Tracht et al [13]. In this study, we applied a susceptible–exposed–infectious/asymptomatic–removed model to estimate *R*_*t*_ and the proportion of asymptomatic individuals in outbreaks happened in China in the past 8 years.

Based on the natural history of influenza [4–5, 9], we established the susceptible–exposed–infectious/asymptomatic–removed (SEIAR) model [9]. The model can be expressed using the following differential equations:

$$\left\{ \begin{array}{l} dS/dt=-\beta S\left(I+\kappa A\right)\\ dE/dt=\beta S\left(I+\kappa A\right)-p\omega \prime E-(1-p)\omega E\\ dI/dt=\left(1-p\right)\omega E-\gamma I\\ dA/dt=p\omega \prime E-\gamma \prime A\\ dR/dt=\gamma I+\gamma \prime A \end{array}\right.$$ (1)

Where *dS/dt*, *dE/dt*, *dI/dt*, *dA/dt*, and *dR/dt* represent the rate of change at moment *t* in the various populations *S*, *E*, *I*, *A*, and *R*, respectively. *β*, *κ*, *ω*, *ω'*, *p*, *γ*, and *γ'* represent the infection rate coefficient, transmission capacity coefficient of *A* in relation to *I*, incubation period coefficient, latent period coefficient, asymptomatic infection ratio, infectious individual removal coefficient, and asymptomatic individual removal coefficient.

### The transmissibility of influenza in outbreaks

Effective reproduction number (*R*_*t*_) is defined as the expected number of secondary infections that result from introducing a single infected individual into an otherwise susceptible population [14–15]. *R*_*t*_ is generally applied to determine the influenza transmission capacity. *R*_*t*_ refers to the number of new disease cases expected to be directly infected by one source of infection during the infection period in the susceptible population [14–15]. If *R*_*t*_<1, the number of infected individuals would decrease toward zero. The disease would not be prevalent and would therefore be gradually eliminated. In contrast, if *R*_*t*_>1, the disease would be prevalent. According to the definition of *R*_*t*_ and the methods reported by Chen et al. [16] and Arino et al. [9], the *R*_*t*_ expression in “model (1)” is as follows:

$$R_{t}=\beta S_{0}\left(\frac{1-p}{\gamma }+\frac{\kappa p}{\gamma^{\prime }}\right)$$ (2)

### Other parameter estimation

Studies have shown that the influenza incubation period, mean latent period, and infection period of asymptomatic individuals are 1–7 days (with a mean of 1.9 days), 1.2 days and 4.2 days, respectively. The infection capacity of asymptomatic individuals is half that of infectious individuals [3–5]. Therefore, the values of the following four parameters were used: *ω* = 0.5263, *ω'* = 0.8333, *γ'* = 0.2439, and *κ* = 0.5. Coefficients *β* and *p* were obtained from model fitting.

### Simulation methods and data analysis

We used SEIAR model to fit the target data (Figure A in S2 File) of all the 613 outbreaks to estimate *β* and *p* per incident. Then_*t*_ of each outbreak was calculated accordingly. Model simulation was conducted using the software Berkeley Madonna 8.3.18 (University of California, Berkeley, Berkeley, USA). Differential equation fitting was performed using the fourth-order Runge-Kutta method with the tolerance set at 0.001. While the curve fit is in progress, Berkeley Madonna displays the root-mean-square deviation between the data and the best fit that has been run.

We analyzed the differences in *p* by subtypes and the differences in *R*_*t*_ by subtypes, provinces, regions, time, location types of infection occurrence, and initial susceptible population sizes. The last item refers to the number of persons affected in an influenza outbreak. For example, in a school, if the cases are limited to one class, then the class size is initial susceptible population size. If the cases happen in more than one class of the same study year, then the total size of all the classes of the same study year will be assumed. Finally, the cases are from different classes, different years, the total number of students in the school will be used. The software SPSS 13.0 was employed to run the data analysis by using *t* test, ANOVA analysis, χ^2^ test and the regression (curve estimation).

### Sensitivity analysis

Of the coefficients in the model, the value *κ* (*κ* = 0.5) was obtained from existing literature [2–4]. But according to recent clinical evidence, the value of *κ* could be low down to 0.1, which suggests that asymptomatic infection might be less important in the spread of epidemics than previously thought [17]. To understand the impact of *κ*, we did a sensitivity analysis based on 10 randomly selected outbreaks in China. Based on the definition of *κ*, we used 0.1–0.9 as *κ* for the sensitivity analysis, and observed the influence of these changes to the *R*_*t*_.

In addition, we analyzed the relationship between *R*_*t*_ and the susceptible population at the beginning of the outbreak (*S*_0_) by using the 10 selected outbreaks. We set 5, 10, 20, 50, 100 and 1000 to do the curve fitting of a same outbreak. A new indicator *p*_*R*_ (where *p*_*R*_ = [*R*_*t*(*S*0)_-*R*_*t*(1000*S*0)_]/*R*_*t*(*S*0)_) was calculated. We then compared *S*_0_ with *p*_*R*_ by using the following the ten functions (Linear, Quadratic, Compound, Growth, Logarithmic, Cubic, S, Exponential, Inverse and Power function) in the software SPSS 13.0.

To consider the robustness of the SEIAR model, and also because four parameters of the SEIAR model, i.e., *ω*, *ω*', *γ*' and *k*, were estimated by references, some uncertainty was present that may affect the results of the constructed models. Sensitivity was evaluated by varying these four parameters, which were divided into 1,000 values ranging from 0.1429 to 1 (indicating incubation periods ranging from 1–7 days), from 0.1429 to 1 (indicating latent periods ranging from 1–7 days), from 0.0833 to 1 (indicating infectious periods of asymptomatic individuals ranging from 1–12 days), and from 0 to 1 (indicating transmissibility of asymptomatic individuals ranging from 0–1 compared to symptomatic individuals), respectively.

## Results

### Basic characteristics of the outbreaks

From April 1, 2005 to November 30, 2013, 2768 ILI emergency public health incidents in China were reported in EPRS. Following the exclusion of incidents with insufficient patient numbers (n = 30), unexamined pathogens, non-influenza viruses, and untyped influenza viruses, a total of 1267 influenza emergency public health incidents were applicable for the following analysis. Of these incidents, 654 incidents without patient data, with incomplete data, or with logical errors were also excluded. The remaining 613 outbreaks were used as the final data in this study. Of these 613 incidents, 138 were A (H3N2) subtype, 68 were A (H1N1) pdm09 subtype, 31 were A (H1N1) subtype, 350 were type B, and 26 were mixed infections (A+B: 6, H1N1+B: 3, H1N1+H3N2: 3, H1N1pdm+A: 1, H1N1pdm+ H1N1: 1, H1N1pdm+H3N2: 1, H1N1pdm+Seasonal: 5, H3N2+B: 6). The two datasets locations of the original 2768 and 613 final outbreaks, despite after tremendous filtering, still resembled to each other.

### The period between disease onset and recovery

In the distributions of the disease course, the longest disease course was 12 days, and the shortest was 1 day; the mean was 4.27 (±1.98 SD), coefficient *γ*was 0.2342.

### Asymptomatic infection ratio

The distributions of *p* of the various subtypes are shown in Table 1. The mean values of *p* of the subtypes ranged from 0.09–0.15, with a minimum of 0 and a maximum of 0.94. The non-parametric Kruskal-Wallis analysis indicated no significant differences in *p* among the subtypes (χ^2^ = 3.117, *P* = 0.538). (Figure B in S2 File)

**Table 1:** Asymptomatic infection ratio distribution of the different influenza virus subtypes.

Subtype	N	Mean	Std. Deviation	Std. Error	95% CI for Mean		Min	Max
					Lower Bound	Upper Bound		
A (H3N2)	138	0.14	0.28	0.02	0.09	0.19	0.00	0.90
A (H1N1) pdm	68	0.09	0.24	0.03	0.03	0.15	0.00	0.89
A (H1N1)	31	0.16	0.28	0.05	0.05	0.26	0.00	0.89
B	350	0.15	0.27	0.01	0.12	0.18	0.00	0.94
Mixed	26	0.12	0.26	0.05	0.01	0.22	0.00	0.85
Total	613	0.14	0.27	0.01	0.12	0.16	0.00	0.94

### *R*_*t*_ of different subtypes

The *R*_*t*_ values of the subtypes are shown in both Figure C in S2 File and Table 2. The mean *R*_*t*_ values of influenza A (H3N2), A (H1N1) pdm, A (H1N1), influenza B, and the mixed types were 8.46, 9.33, 7.63, 7.79, and 10.03, respectively. The homogeneity of variance test resulted in a Levene value of 1.243 (*P* = 0.291). Therefore, the variances of *R*_*t*_ of the subtypes were considered homogeneous. Moreover, an analysis of variance was used for the analysis among the subtypes, resulting in *F* = 2.051 and *P* = 0.086. Therefore, differences in *R*_*t*_ between the subtypes were considered no statistical significance.

**Table 2:** Distributions of *R*_*t*_ of the variables.

Variable	N	Mean	Std. Deviation	Std. Error	95% CI for Mean		Min	Max
					Lower Bound	Upper Bound		
Subtype								
A (H3N2)	138	8.46	5.88	0.50	7.47	9.45	1.17	37.80
A (H1N1) pdm	68	9.33	5.48	0.66	8.00	10.66	1.18	32.59
A (H1N1)	31	7.63	4.20	0.75	6.09	9.17	2.64	22.53
B	350	7.79	5.24	0.28	7.24	8.34	1.19	43.64
Mixed	26	10.03	8.44	1.66	6.62	13.44	3.21	43.96
Region								
Northern	129	7.64	5.52	0.49	6.68	8.61	1.45	43.96
Southern	484	8.35	5.56	0.25	7.85	8.84	1.17	43.64
Venue								
Northern Region								
Kindergarten	1	4.39	NA	NA	NA	NA	4.39	4.39
Primary school	79	8.19	6.64	0.75	6.7	9.67	1.45	43.96
Primary + middle school	2	4.61	0.69	0.49	-1.62	10.84	4.12	5.1
Middle + high school	37	6.92	2.97	0.49	5.93	7.91	2.35	14.28
Technical secondary school	2	7.13	0.62	0.44	1.54	12.72	6.69	7.57
College	2	6.6	6.62	4.68	-52.87	66.07	1.92	11.28
Other school	3	5.29	1.48	0.85	1.62	8.96	3.98	6.89
Work place	2	9.24	1.22	0.87	-1.76	20.23	8.37	10.1
Community	1	7.66	NA	NA	NA	NA	7.66	7.66
Southern Region								
Kindergarten	4	6.33	3.94	1.97	0.06	12.6	2.6	11.67
Primary school	265	8.4	5.9	0.36	7.69	9.11	1.17	43.64
Primary + middle school	3	7.39	2.79	1.61	0.47	14.31	4.58	10.15
Middle + high school	154	7.83	4.45	0.36	7.12	8.54	1.18	27.92
Technical secondary school	6	11.31	5.5	2.24	5.54	17.08	6.51	21.34
College	4	8.14	2.54	1.27	4.11	12.19	6.03	11.83
Other school	13	10.5	7.85	2.18	5.75	15.25	3.35	32.59
Work place	25	9.43	7.21	1.44	6.45	12.4	2.61	31.76
Community	10	8.78	5.98	1.89	4.5	13.05	3.07	22.53
Population								
Northern Region								
0~	74	7.9	5.19	0.6	6.7	9.11	2.35	32.53
1000~	25	7.34	3.61	0.72	5.85	8.83	2.86	16.89
2000~	30	7.26	7.45	1.36	4.48	10.04	1.45	43.96
Southern Region								
0~	216	9.55	6.6	0.45	8.66	10.43	1.19	43.64
1000~	141	7.23	4.42	0.37	6.5	7.97	1.48	32.53
2000~	127	7.54	4.25	0.38	6.8	8.29	1.17	27.62
Year								
Northern Region								
2005	2	10.29	6.47	3.87	-38.82	59.39	6.42	14.15
2006	46	6.7	3.46	0.51	5.68	7.73	2.35	17.4
2007	23	5.96	2.42	0.51	4.91	7.01	2.85	11.54
2008	3	11.24	8.08	4.66	-8.82	31.31	4.85	20.32
2009	39	8.77	7.96	1.27	6.19	11.35	1.92	43.96
2010	4	9.18	7.88	3.94	-3.36	21.72	1.45	19.74
2011	2	8.41	4.06	2.87	-28.06	44.88	5.54	11.28
2012	8	8.2	4.66	1.65	4.3	12.09	3.26	18.07
2013	2	12.63	6.02	4.26	-41.5	66.76	8.37	16.89
Southern Region								
2005	28	5.96	2.96	0.56	4.82	7.11	1.19	14.06
2006	100	7.64	4.9	0.49	6.67	8.62	2.26	32.53
2007	44	9.49	7.74	1.17	7.14	11.85	1.48	39.66
2008	29	5.51	2.74	0.51	4.47	6.55	2.15	16.78
2009	175	9.09	5.45	0.41	8.28	9.9	1.17	37.8
2010	12	9.35	5.33	1.54	5.97	12.74	4.12	18.55
2011	35	9.8	7.89	1.33	7.09	12.51	1.18	43.64
2012	30	7.7	4.06	0.74	6.19	9.22	1.88	17.22
2013	31	8.19	5.13	0.92	6.31	10.07	3.32	30.43
Season								
Northern Region								
1	30	7.82	4.93	0.9	5.97	9.66	1.45	20.32
2	71	6.89	4.58	0.54	5.8	7.97	2.35	32.53
3	7	7.47	3.57	1.35	4.17	10.77	1.92	12.68
4	21	10.01	8.63	1.88	6.09	13.94	2.44	43.96
Southern Region								
1	145	7.45	4.73	0.39	6.67	8.22	1.18	32.53
2	148	8.42	5.47	0.45	7.53	9.31	1.17	39.66
3	88	9.76	6.36	0.68	8.41	11.1	1.57	37.8
4	103	8.31	5.88	0.58	7.16	9.46	1.19	43.64
Total	613	8.20	5.56	0.22	7.76	8.64	1.17	43.96

NA, not available.

### *R*_*t*_ in southern and northern regions of China

Of the 613 incidents, 129 from the northern regions in China exhibited a mean *R*_*t*_ of 7.64 (±5.52), and the remaining 484 incidents from the southern regions exhibited a mean *R*_*t*_ of 8.35 (±5.56). The *t*-test indicated that the difference in the *R*_*t*_ value between the northern and southern regions was not statistically significant (*t* = -1.278, *P* = 0.202; Table 2).

### *R*_*t*_ in different provinces

Distributions of *R*_*t*_ in different provinces are shown in Table A in S2 File. There were 613 outbreaks distributed in 26 provinces in Mainland China (Hong Kong SAR, Macao SAR, and Taiwan were not included). Of these provinces, Shanxi, Shaanxi, and Hainan provinces exhibited the largest *R*_*t*_ values of 13.07, 13.07, and 10.60, respectively. The autonomous regions of Inner Mongolia, Ningxia and Shanghai city exhibited the smallest *R*_*t*_ values of 5.15, 5.73, and 5.80, respectively. Non-parametric Kruskal-Wallis analysis indicated significant differences in the *R*_*t*_ values among the provinces (χ^2^ = 48.358, *P* = 0.003).

### *R*_*t*_ at different locations

Of the 613 incidents, primary (344 incidents) and secondary (191 incidents) schools accounted for 87.28% of the total incidents. The homogeneity of variance test resulted in a Levene value of 1.373 (*P* = 0.205). Therefore, the variances in *R*_*t*_ of the subtypes were considered homogeneous, and analysis of variance was used for the analysis among the subtypes. Analysis of variance resulted in *F* = 0.866 and *P* = 0.545, indicating that the differences in *R*_*t*_ among the subtypes were not statistically significant (Table 2). A total of 129 and 484 outbreaks took place in northern and southern China respectively. Although there was a difference in the number of outbreaks, there was no significant difference in *R*_*t*_ for different regions (Northern: *F* = 0.379 and *P* = 0.930 by analysis of variance, Southern: *F* = 0.823 and *P* = 0.583).

### *R*_*t*_ in various populations

As shown in Table 2, of the 613 incidents, 290 incidents with an affected number of less than 1000 were designated “0~ group”. Similarly, 166 incidents with an affected number of 1000–1999 and the remaining 157 incidents with an affected number of ≥2000 were designated “1000~ group” and “2000~ group”, respectively. The homogeneity of variance test resulted in a Levene value of 6.556 (*P* = 0.002), indicating that the variances of *R*_*t*_ at the various population levels were heterogeneous. Following mixed sorting of *R*_*t*_, we conducted a homogeneity of variance test for the *R*_*t*_ order. The result indicated that the Levene value was 0.032 (*P* = 0.969), suggesting that the variances of the *R*_*t*_ order at the various population levels were homogeneous, and analysis of variance was conducted. The analysis of variance resulted in a difference between the population levels that was statistically significant (*F* = 6.972, *P* = 0.001). Bonferroni paired comparisons indicated that the differences between the “0~ group” and “1000~ group” and between the “0~ group” and “2000~ group” were statistically significant and that the difference between the “1000~ group” and “2000~ group” was not statistically significant. In the northern region, again there was no significant difference in *R*_*t*_ for different population size (*F* = 0.190, *P* = 0.827). In contrast, it was for the southern part (*F* = 9.457, *P* = 0.000). Post-hoc analysis with Bonferroni adjustments showed that cities with small population (0~ group) had significantly higher *R*_*t*_ than their counterparts.

### *R*_*t*_ at different times of occurrence

As shown in Table 2, the epidemics were prevalent every year, with the most incidents occurring in 2009 (214), followed by 2006 (146), whereas 2010 had the least number of incidents (16). The non-parametric Kruskal-Wallis analysis resulted in differences in *R*_*t*_ between the different years that were statistically significant (χ^2^ = 27.955, *P* = 0.000). The 2009 flu pandemic appeared to strike more in the southern region, because analysis of variance showed that there was no significant difference in *R*_*t*_ for different years (*F* = 1.121, *P* = 0.354) while there was for the southern part between 2008 and 2009 (*F* = 2.897, *P* = 0.004).

All 613 incidents were distributed across all four seasons, with more incidents in the second season than those in the first or fourth seasons where the first to the fourth seasons are the same as spring, summer, fall and winter. The third season had the least number of incidents (Table 2). The homogeneity of variance test resulted in a Levene value of 1.915 (*P* = 0.126). Therefore, the variances of *R*_*t*_ in the various seasons were homogenous, and analysis of variance was used to analyze the subtypes. The analysis of variance resulted in differences in *R*_*t*_ between the different seasons that were statistically significant (*F* = 3.306, *P* = 0.020). The Bonferroni paired comparison resulted in a statistically significant difference between the first and third seasons, whereas the differences between the remaining seasons were not statistically significant. Influenza seasonality were not found to result in different *R*_*t*_ in the northern region (*F* = 1.777, *P* = 0.155). In contrast, in the southern counterpart, there was lower *R*_*t*_ in the first season and higher *R*_*t*_ in the third season.

### Sensitivity analysis

Among the 10 randomly selected outbreaks, two located in Fujian province, the others located in Inner Mongolia, Gansu, Jiangxi, Guangdong, Zhejiang, Jiangsu, Guangxi, Hubei, respectively. Four of them were occurred in the year 2006, three in 2007, one in 2008, one in 2009 and one in 2013. According to the results of the sensitivity analysis, we found that *κ* and *S*_0_ can only slightly impact *R*_*t*_ (see Tables 3 and 4). The larger *S*_0_ is, the smaller *p*_*R*_ would be. The results of curve estimation showed that the 10 functions could depicted the relationship between *S*_0_ and *p*_*R*_. But according to the formula of the functions, the fuction Inverse (*p*_*R*_ = 1.344+3841.5/*S*_0_) should be the best one to estimate, of which the coefficient of determination *R*^2^ = 0.776, *P* = 0.001. For the outbreak which has a relative larger *S*_0_ (e.g. outbreak ID = 25), *R*_*t*_ would be stable after 50*S*_0_, but along with the decreasing of *S*_0_, *R*_*t*_ would be stable after 100*S*_0_ (e.g. outbreak ID = 215).

**Table 3:** Estimated *R*_*t*_ value based on different *κ* in 10 randomly selected outbreaks in China.

Outbreak ID	Province	Year	*R*_*t*_								
			*κ* = 0.9	*κ* = 0.8	*κ* = 0.7	*κ* = 0.6	*κ* = 0.5	*κ* = 0.4	*κ* = 0.3	*κ* = 0.2	*κ* = 0.1
1	Inner Mongolia	2007	3.88	3.89	3.91	3.93	3.97	4.00	4.04	4.10	4.16
13	Fujian	2007	6.96	6.97	6.97	6.97	6.98	7.00	7.03	7.07	7.12
25	Gansu	2007	5.24	5.27	5.30	5.33	5.38	5.43	5.49	5.57	5.66
103	Fujian	2008	4.83	4.84	4.86	4.87	4.89	4.92	4.96	5.01	5.06
215	Jiangxi	2006	6.82	6.82	6.83	6.84	6.87	6.89	6.93	6.99	7.05
245	Guangdong	2006	6.13	6.13	6.14	6.15	6.17	6.19	6.22	6.27	6.33
251	Zhejiang	2006	2.56	2.57	2.59	2.61	2.63	2.66	2.69	2.73	2.78
275	Jiangsu	2006	7.06	7.06	7.08	7.11	7.14	7.18	7.24	7.30	7.39
475	Guangxi	2013	6.34	6.34	6.33	6.33	6.33	6.34	6.35	6.37	6.41
533	Hubei	2009	6.24	6.24	6.25	6.25	6.27	6.29	6.32	6.36	6.42

**Table 4:** Estimated *R*_*t*_ value based on different *S*_0_ in 10 randomly selected outbreaks in China.

Outbreak ID	*S*_0_	*p*_*R*_ (%)	*R*_*t*_						
			*S*_0_	5*S*_0_	10*S*_0_	20*S*_0_	50*S*_0_	100*S*_0_	1000*S*_0_
103	295	13.18	4.89	4.35	4.3	4.27	4.26	4.25	4.25
215	508	8.96	6.87	6.35	6.3	6.27	6.26	6.25	6.25
275	595	11.73	7.14	6.44	6.37	6.34	6.32	6.31	6.31
533	747	5.02	6.27	6.01	5.98	5.97	5.96	5.96	5.95
1	930	6.9	3.97	3.74	3.71	3.7	3.7	3.69	3.69
13	957	3.92	6.98	6.76	6.73	6.72	6.71	6.71	6.71
475	2441	1.04	6.33	6.27	6.27	6.26	6.26	6.26	6.26
245	2848	1.49	6.17	6.1	6.09	6.09	6.08	6.08	6.08
251	3079	1.57	2.63	2.6	2.59	2.59	2.59	2.59	2.59
25	3624	5.19	5.38	5.15	5.12	5.11	5.1	5.1	5.1

Our model was sensitive to the parameters *ω*, *ω'*, and *k*; however, the value that we set in our model led to a similar prevalence as the mean value of sensitivity analysis based on the constructed models (Figure D Panels A-C in S2 File). The model is not sensitive to the parameter *γ'*, and the prevalence was highly similar to the mean, mean—SD, mean + SD, or our set value (Figure D Panel D in S2 File).

## Discussion

This study is the first to systematically analyze the influenza outbreaks in Mainland China by ODE modeling.

We estimated the proportion of the asymptomatic in influenza outbreaks, as well as the basic reproduction numbers under different groups of influenza virus subtypes, northern and southern regions, provinces, sites, size of the population affected, years and seasons. The data provide China and also the rest of the world a reference to understand characteristics of transmission and develop prevention and control strategies.

The existence of asymptomatic cases significantly challenges the prevention and control of influenza. Some studies have shown that the *p* value of influenza can exceed 75%, and the difference between seasonal and pandemic influenza was not significant [2]. But recently, Leung et al [18] did a systematic review and found that estimates of the *p* based on outbreak investigations and household transmission studies appeared to provide more homogeneity in estimates of the *p*, with most point estimates in the range 4%–28% and a pooled mean of 16% (95% CI: 13–19). Our study indicated that the *p* value of the influenza virus can reach a maximum of 94% and exhibited a mean of 14% (95% CI: 12%–16%) during an outbreak, which was similar to the finding of Leung et al [18]; however, most outbreaks did not include asymptomatic infections. Different subtypes appeared to follow similar transmission pattern and this information can be exploited to strategically allocate resources in future prevention and control management. Our estimated *p* were different from what has been found in other studies, likely due to that the source of our data is from large-scale prevalence data from schools. We only included the incident with more than or equal to 30 cases of ILI per week, which intrinsically leads to lower proportion of asymptomatic individuals.

According to the definition of *R*_*t*_, influenza spreads more rapidly with a larger *R*_*t*_, resulting in greater challenges in the prevention and control of an epidemic. The results of this study indicated that during an influenza outbreak, the transmission capacity of the influenza virus was relatively high; the mean *R*_*t*_ reached 8.20 (95% CI: 7.76–8.64), whereas *R*_*t*_ of the total population is approximately 1.2–2.3 [11, 14, 19]. This difference may be related to the high population density and high population contact level in schools, collective organizations, closed locations, and certain small villages. Our estimated *R*_*t*_ were also different from what has been found in other studies, likely due to the inclusion criteria aforementioned, which means that the data we collected would be super spreading event and this intrinsically leads to higher *R*_*t*_. Indeed, a study in Taiwan also showed that *R*_*t*_ estimated from influenza outbreaks in schools can be up to 19 [16].

According to these results, transmission capacity differences between the subtypes were not significant, indicating that outbreaks caused by seasonal influenza A (H3N2), A (H1N1), and A (H1N1) pdm09 viruses may be handled using similar prevention and control measures and do not require different treatments. This finding provides important scientific basis for the future prevention and control of influenza outbreaks in China. This result also quantitatively supported the decision by Chinese health administrative departments to adjust influenza A (H1N1) pdm09 from Category B to Category C and to combine influenza A (H1N1) pdm09 with current influenza management [20].

In spite of extensive data collection and analyses, this study carried limitations. First, we only included the incident with more than or equal to 30 cases of ILI per week. Second, data uncertainty such as reporting errors and under-reporting could lead to estimation errors of varying size.

Our study also indicated that there were no statistically significant differences in influenza transmission capacity during an outbreak between northern and southern regions in China or different location types in which incidents occurred. Although the difference between provinces was significant, this difference may arise from the small or zero numbers of influenza outbreaks reported by certain provinces. Notably, influenza transmission capacity is related to various population levels. The difference between the numbers of affected people was significant between the “0~ group” and “1000~ group” and between the “0~ group” and “2000~ group”. If the sample was less than 1000 people, the influenza transmission capacity was higher than a sample of ≥1000 people, indicating that schools, prisons, and factories with smaller populations have relatively higher prevention and control measures during an influenza outbreak and should apply earlier and more substantial prevention and control measures. Additionally, the influenza transmission capacity exhibits seasonality. The difference between the first and the third seasons was statistically significant, whereas the difference between the remaining seasons was not statistically significant. Transmission capacity in the third season was stronger than that in the first season. Therefore, future prevention and control measures for outbreaks should be conducted throughout the year in Mainland China, particularly during the third season. Furthermore, our study provides important prevention and control indicators for other countries.

## Supporting Information

S1 File Interval from illness onset to recovery dates of 283 influenza cases. (PDF)

S2 File **This file contains the following: Figure A. Temporal distribution of new influenza cases in a school in Mainland China, October 2009. Figure B. Asymptomatic infection ratio of various influenza virus subtypes**. Figures 2-A to 2-F show the distribution of asymptomatic infection ratios of all of the subtypes of influenza (total, A (H3N2), A (H1N1) pdm, A (H1N1), B, and mixed subtypes, respectively). **Figure C. Histogram of which means that the data we collected would be super spreading event of the influenza virus subtypes**. Figures 3-A to 3-F show the distribution of *R*_*t*_ of all of the subtypes of influenza (total, A (H3N2), A (H1N1) pdm, A (H1N1), B, and mixed subtypes, respectively). **Figure D. Results of the sensitivity analysis of the SEIAR model**. Figures 4-A to 4-D show the results of the sensitivity analysis of *κ*, *ω*, *ω'*, and*γ'*, respectively. **Table A. Distribution of *R***_***t***_ **in various provinces**.
(DOC)
