# Supplementary material for: Transmissibility of the Influenza Virus during Influenza Outbreaks and Related Asymptomatic Infection in Mainland China, 2005-2013
Source: PLoS One. 2016 Nov 23;11(11):e0166180. doi: 10.1371/journal.pone.0166180 (PMC5120824; doi:10.1371/journal.pone.0166180)
Supplement: S1 File — (PDF) [file pone.0166180.s001.pdf]

ID	Subtype	Interval
1	B	1
2	B	1
3	B	1
4	B	1
5	B	1
6	B	2
7	B	2
8	B	3
9	B	3
10	B	3
11	B	3
12	B	3
13	B	4
14	B	4
15	B	5
16	B	5
17	B	5
18	B	5
19	B	5
20	B	5
21	B	6
22	B	7
23	H1N1	6
24	H1N1	6
25	H1N1	6
26	H1N1	7
27	H1N1	7
28	H1N1	7
29	H1N1	7
30	H1N1	10
31	H1N1	10
32	H1N1	2
33	H1N1	2
34	H1N1	2
35	H1N1	2
36	H1N1	3
37	H1N1	3
38	H1N1	3
39	H1N1	3
40	H1N1	3
41	H1N1	3
42	H1N1	3

43	H1N1	3
44	H1N1	3
45	H1N1	3
46	H1N1	3
47	H1N1	3
48	H1N1	4
49	H1N1	4
50	H1N1	4
51	H1N1	4
52	H1N1	4
53	H1N1	4
54	H1N1	4
55	H1N1	4
56	H1N1	4
57	H1N1	4
58	H1N1	4
59	H1N1	4
60	H1N1	4
61	H1N1	4
62	H1N1	4
63	H1N1	4
64	H1N1	4
65	H1N1	5
66	H1N1	5
67	H1N1	5
68	H1N1	5
69	H1N1	5
70	H1N1	5
71	H1N1	5
72	H1N1	5
73	H1N1	5
74	H1N1	5
75	H1N1	5
76	H1N1	5
77	H1N1	5
78	H1N1	5
79	H1N1	5
80	H1N1	5
81	H1N1	5
82	H1N1	5
83	H1N1	5
84	H1N1	5
85	H1N1	5

86	H1N1	6
87	H1N1	6
88	H1N1	6
89	H1N1	6
90	H1N1	6
91	H1N1	6
92	H1N1	6
93	H1N1	6
94	H1N1	6
95	H1N1	6
96	H1N1	6
97	H1N1	6
98	H1N1	7
99	H1N1	7
100	H1N1	7
101	H1N1	7
102	H1N1	7
103	H1N1	7
104	H1N1	8
105	H1N1	9
106	H1N1	9
107	H1N1	9
108	H1N1	9
109	H1N1	9
110	H1N1	10
111	H1N1	10
112	H1N1	5
113	H1N1	5
114	H1N1	5
115	H1N1	5
116	H1N1	5
117	H1N1	5
118	H1N1	5
119	H1N1	5
120	H1N1	5
121	H1N1	5
122	H1N1	5
123	H1N1	5
124	H1N1	5
125	H1N1	5
126	H1N1	5
127	H1N1	5
128	H1N1	5

129	H1N1	5
130	H1N1	5
131	H1N1	5
132	H1N1	5
133	H1N1	5
134	H3N2	4
135	H3N2	4
136	H3N2	4
137	H3N2	5
138	H3N2	5
139	H3N2	5
140	H3N2	5
141	H3N2	5
142	H3N2	5
143	H3N2	6
144	H3N2	6
145	H3N2	7
146	H3N2	7
147	H3N2	7
148	H3N2	7
149	H3N2	8
150	H3N2	8
151	H3N2	8
152	H3N2	8
153	H3N2	12
154	H3N2	2
155	H3N2	2
156	H3N2	4
157	H3N2	4
158	H3N2	5
159	H3N2	5
160	H3N2	5
161	H3N2	5
162	H3N2	5
163	H3N2	8
164	H3N2	8
165	H3N2	10
166	H3N2	1
167	H3N2	1
168	H3N2	1
169	H3N2	1
170	H3N2	1
171	H3N2	1

172	H3N2	1
173	H3N2	1
174	H3N2	1
175	H3N2	1
176	H3N2	1
177	H3N2	1
178	H3N2	1
179	H3N2	1
180	H3N2	1
181	H3N2	2
182	H3N2	2
183	H3N2	2
184	H3N2	2
185	H3N2	2
186	H3N2	2
187	H3N2	2
188	H3N2	2
189	H3N2	2
190	H3N2	2
191	H3N2	2
192	H3N2	2
193	H3N2	2
194	H3N2	2
195	H3N2	2
196	H3N2	2
197	H3N2	2
198	H3N2	3
199	H3N2	3
200	H3N2	3
201	H3N2	3
202	H3N2	3
203	H3N2	3
204	H3N2	3
205	H3N2	4
206	H3N2	4
207	H3N2	4
208	H3N2	6
209	H3N2	7
210	H3N2+B	2
211	H3N2+B	2
212	H3N2+B	2
213	H3N2+B	2
214	H3N2+B	3

215	H3N2+B	3
216	H3N2+B	3
217	H3N2+B	3
218	H3N2+B	3
219	H3N2+B	3
220	H3N2+B	3
221	H3N2+B	3
222	H3N2+B	3
223	H3N2+B	3
224	H3N2+B	3
225	H3N2+B	3
226	H3N2+B	3
227	H3N2+B	3
228	H3N2+B	3
229	H3N2+B	3
230	H3N2+B	3
231	H3N2+B	3
232	H3N2+B	3
233	H3N2+B	3
234	H3N2+B	3
235	H3N2+B	3
236	H3N2+B	3
237	H3N2+B	3
238	H3N2+B	3
239	H3N2+B	3
240	H3N2+B	3
241	H3N2+B	3
242	H3N2+B	3
243	H3N2+B	3
244	H3N2+B	4
245	H3N2+B	4
246	H3N2+B	4
247	H3N2+B	4
248	H3N2+B	4
249	H3N2+B	4
250	H3N2+B	4
251	H3N2+B	4
252	H3N2+B	4
253	H3N2+B	4
254	H3N2+B	4
255	H3N2+B	4
256	H3N2+B	4
257	H3N2+B	4

258	H3N2+B	4
259	H3N2+B	4
260	H3N2+B	4
261	H3N2+B	4
262	H3N2+B	4
263	H3N2+B	4
264	H3N2+B	4
265	H3N2+B	4
266	H3N2+B	4
267	H3N2+B	4
268	H3N2+B	4
269	H3N2+B	4
270	H3N2+B	4
271	H3N2+B	4
272	H3N2+B	4
273	H3N2+B	5
274	H3N2+B	5
275	H3N2+B	5
276	H3N2+B	5
277	H3N2+B	5
278	H3N2+B	5
279	H3N2+B	5
280	H3N2+B	5
281	H3N2+B	5
282	H3N2+B	5
283	H3N2+B	6
