# Supplementary material for: Transmissibility of the Influenza Virus during Influenza Outbreaks and Related Asymptomatic Infection in Mainland China, 2005-2013
Source: PLoS One. 2016 Nov 23;11(11):e0166180. doi: 10.1371/journal.pone.0166180 (PMC5120824; doi:10.1371/journal.pone.0166180)
Supplement: S2 File — This file contains the following: Figure A. Temporal distribution of new influenza cases in a school in Mainland China, October 2009. Figure B. Asymptomatic infection ratio of various influenza virus subtypes. Figures 2-A to 2-F show the distribution of asymptomatic infection ratios of all of the subtypes of influenza (total, A (H3N2), A (H1N1) pdm, A (H1N1), B, and mixed subtypes, respectively). Figure C. Histogram of which means that the data we collected would be super spreading event of the influenza virus subtypes. Figures 3-A to 3-F show the distribution of Rt of all of the subtypes of influenza (total, A (H3N2), A (H1N1) pdm, A (H1N1), B, and mixed subtypes, respectively). Figure D. Results of the sensitivity analysis of the SEIAR model. Figures 4-A to 4-D show the results of the sensitivity analysis of κ, ω, ω', andγ', respectively. Table A. Distribution of Rt in various provinces. (DOC) [file pone.0166180.s002.doc]

**S1 Fig. Temporal distribution of new influenza cases in a school in Mainland China, October 2009.**

**
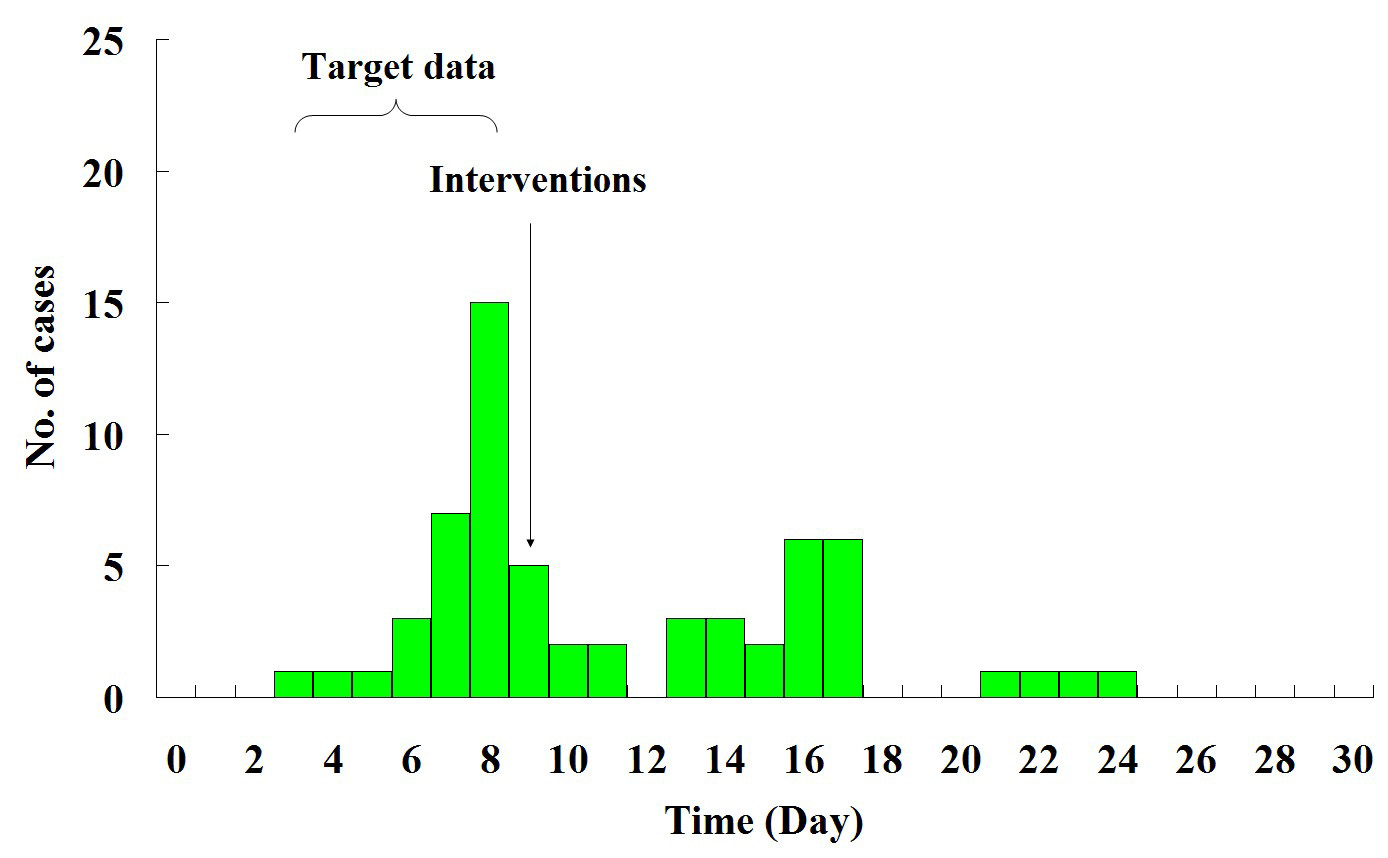
**

**S2 Fig. Asymptomatic infection ratio of various influenza virus subtypes. S2** Fig A to F show the distribution of asymptomatic infection ratios of all of the subtypes of influenza (total, A (H3N2), A (H1N1) pdm, A (H1N1), B, and mixed subtypes, respectively).


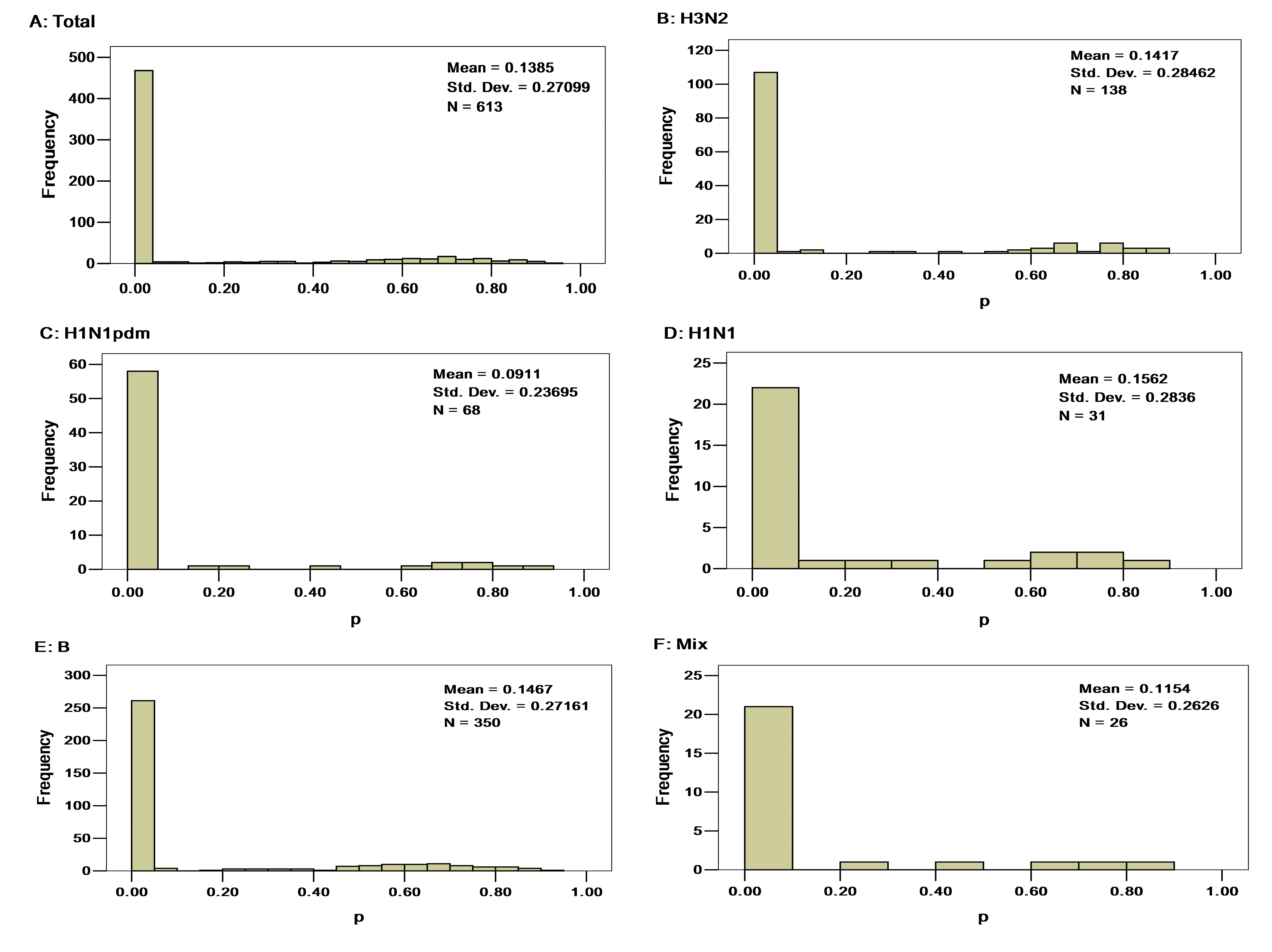


**S3 Fig. Histogram of *R*_0_ of the influenza virus subtypes.** S3 Figs A to F show the distribution of *R*_0_ of all of the subtypes of influenza (total, A (H3N2), A (H1N1) pdm, A (H1N1), B, and mixed subtypes, respectively).


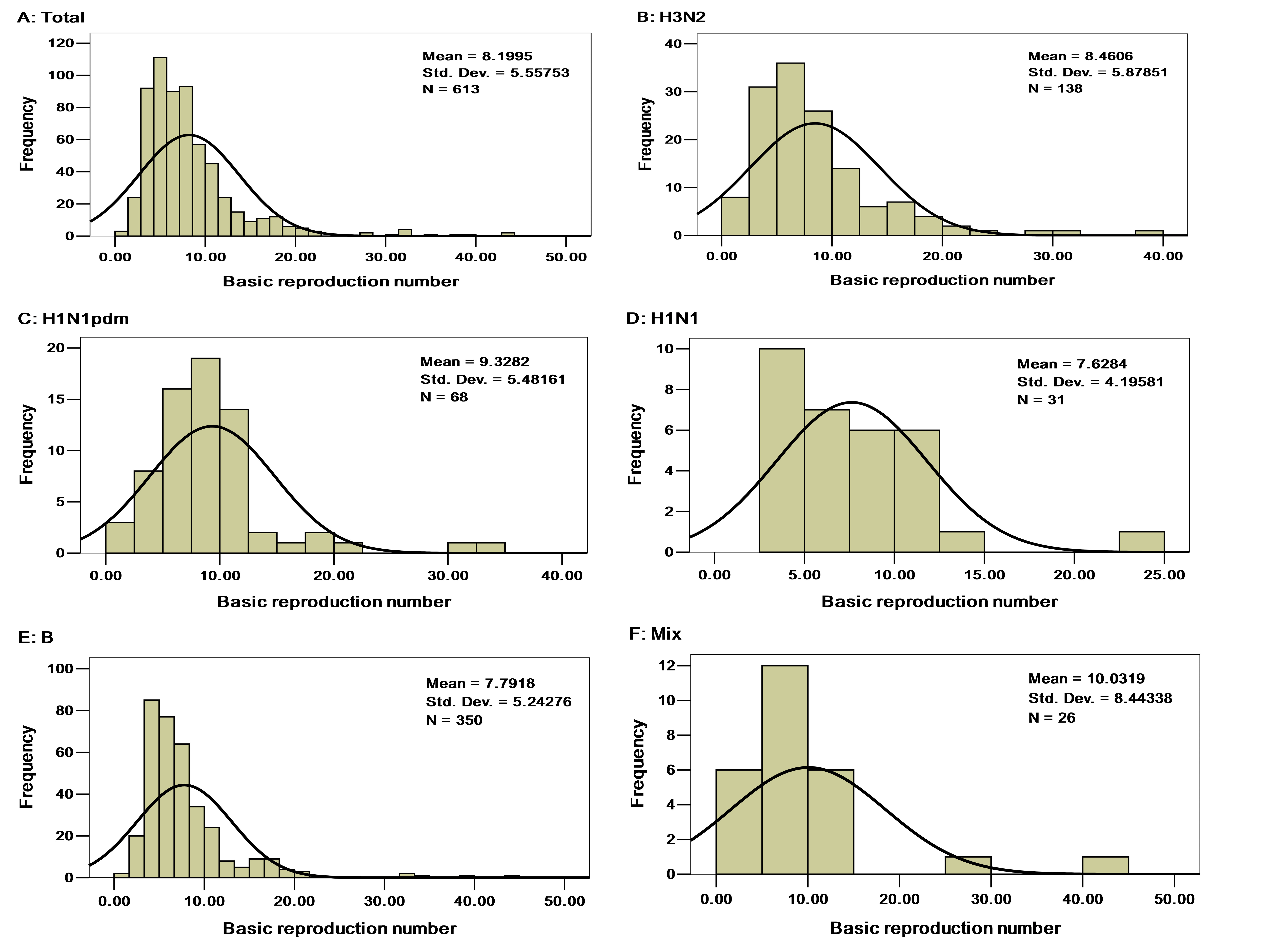


**S4 Fig. Results of the sensitivity analysis of the SEIAR model.** S4 Fig A to D show the results of the sensitivity analysis of *κ*, *ω*, *ω'*, and*γ'*, respectively.


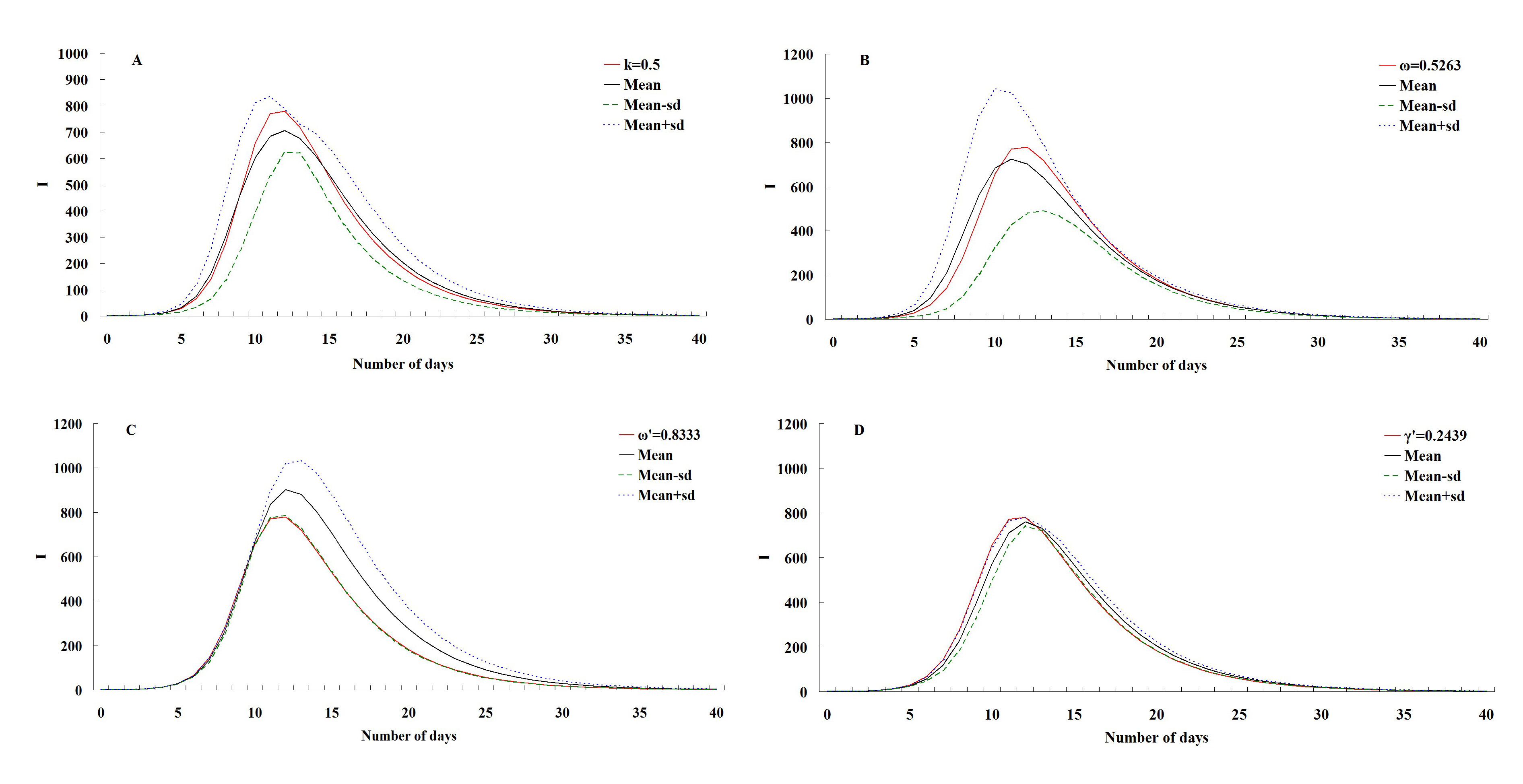
**S1 Table. Distribution of *R*_0_ in various provinces.**

| Province | N | Mean | Std. Deviation | Std. Error | 95% CI for Mean | | Min | Max |
| --- | --- | --- | --- | --- | --- | --- | --- | --- |
|  |  |  |  |  | Lower Bound | Upper Bound |  |  |
| Anhui | 6 | 7.71 | 3.14 | 1.28 | 4.41 | 11.00 | 4.58 | 11.83 |
| Beijing | 2 | 6.91 | 2.67 | 1.89 | -17.05 | 30.86 | 5.02 | 8.79 |
| Fujian | 14 | 5.81 | 3.19 | 0.85 | 3.97 | 7.65 | 1.17 | 11.44 |
| Gansu | 34 | 6.65 | 3.37 | 0.58 | 5.47 | 7.82 | 2.44 | 19.74 |
| Guangdong | 80 | 7.04 | 4.88 | 0.55 | 5.95 | 8.12 | 1.19 | 32.59 |
| Guangxi | 56 | 9.31 | 8.34 | 1.12 | 7.08 | 11.54 | 1.18 | 43.64 |
| Guizhou | 39 | 8.83 | 4.85 | 0.78 | 7.26 | 10.40 | 1.79 | 21.21 |
| Hainan | 2 | 10.60 | 1.48 | 1.05 | -2.68 | 23.87 | 9.55 | 11.64 |
| Hebei | 10 | 6.02 | 3.21 | 1.02 | 3.72 | 8.32 | 2.44 | 12.68 |
| Henan | 10 | 8.72 | 4.76 | 1.51 | 5.31 | 12.13 | 3.65 | 19.64 |
| Hubei | 40 | 9.75 | 5.42 | 0.86 | 8.02 | 11.49 | 2.85 | 27.62 |
| Hunan | 32 | 9.14 | 4.84 | 0.86 | 7.40 | 10.89 | 3.24 | 20.78 |
| Jiangsu | 64 | 7.99 | 3.74 | 0.47 | 7.06 | 8.93 | 2.98 | 18.38 |
| Jiangxi | 12 | 8.77 | 5.06 | 1.46 | 5.55 | 11.99 | 3.40 | 19.72 |
| Inner Mongolia | 13 | 5.15 | 1.85 | 0.51 | 4.03 | 6.27 | 2.35 | 8.45 |
| Ningxia | 2 | 5.80 | 2.50 | 1.77 | -16.69 | 28.29 | 4.03 | 7.57 |
| Shandong | 6 | 6.93 | 4.22 | 1.72 | 2.50 | 11.35 | 4.39 | 15.08 |
| Shanxi | 10 | 13.07 | 11.84 | 3.74 | 4.60 | 21.54 | 4.18 | 43.96 |
| Shaanxi | 9 | 13.07 | 8.92 | 2.97 | 6.22 | 19.93 | 3.32 | 32.53 |
| Shanghai | 2 | 5.73 | 1.27 | 0.90 | -5.71 | 17.17 | 4.83 | 6.63 |
| Sichuan | 5 | 6.45 | 3.56 | 1.59 | 2.03 | 10.87 | 2.31 | 10.50 |
| Tianjin | 23 | 6.94 | 4.03 | 0.84 | 5.20 | 8.68 | 1.45 | 20.32 |
| Xinjiang | 8 | 7.48 | 3.17 | 1.12 | 4.83 | 10.12 | 3.26 | 12.34 |
| Yunnan | 48 | 9.42 | 6.49 | 0.94 | 7.53 | 11.30 | 3.32 | 37.80 |
| Zhejiang | 61 | 7.92 | 6.23 | 0.80 | 6.32 | 9.52 | 1.48 | 32.53 |
| Chongqing | 25 | 7.81 | 3.46 | 0.69 | 6.38 | 9.23 | 3.23 | 17.45 |
| Qinghai | 0 | NA | NA | NA | NA | NA | NA | NA |
| Tibet | 0 | NA | NA | NA | NA | NA | NA | NA |
| Heilongjiang | 0 | NA | NA | NA | NA | NA | NA | NA |
| Jilin | 0 | NA | NA | NA | NA | NA | NA | NA |
| Liaoning | 0 | NA | NA | NA | NA | NA | NA | NA |
| Taiwan | NA | NA | NA | NA | NA | NA | NA | NA |
| Hong Kong | NA | NA | NA | NA | NA | NA | NA | NA |
| Macao | NA | NA | NA | NA | NA | NA | NA | NA |
| Total | 613 | 8.20 | 5.56 | 0.22 | 7.76 | 8.64 | 1.17 | 43.96 |
